# Racial, Ethnic, and Socioeconomic Inequities in Glucagon-Like Peptide-1 Receptor Agonist Use Among Patients With Diabetes in the US

**DOI:** 10.1001/jamahealthforum.2021.4182

**Published:** 2021-12-17

**Authors:** Lauren A. Eberly, Lin Yang, Utibe R. Essien, Nwamaka D. Eneanya, Howard M. Julien, Jing Luo, Ashwin S. Nathan, Sameed Ahmed M. Khatana, Elias J. Dayoub, Alexander C. Fanaroff, Jay Giri, Peter W. Groeneveld, Srinath Adusumalli

**Affiliations:** 1 Cardiovascular Division, Perelman School of Medicine at the University of Pennsylvania, Philadelphia; 2 Center for Cardiovascular Outcomes, Quality, and Evaluative Research, University of Pennsylvania, Philadelphia; 3 Penn Cardiovascular Center for Health Equity and Social Justice, University of Pennsylvania, Philadelphia; 4 Leonard Davis Institute of Health Economics, University of Pennsylvania, Philadelphia; 5 Division of General Internal Medicine, University of Pittsburgh School of Medicine, Pittsburgh, Pennsylvania; 6 Center for Health Equity Research and Promotion, Veterans Affairs Pittsburgh Healthcare System, Pittsburgh, Pennsylvania; 7 Renal-Electrolyte and Hypertension Division, Perelman School of Medicine at the University of Pennsylvania, Philadelphia; 8 Corporal Michael J. Crescenz Veterans Affairs Medical Center, Philadelphia, Pennsylvania; 9 Division of General Internal Medicine, Perelman School of Medicine at the University of Pennsylvania, Philadelphia

## Abstract

**Importance:**

Randomized clinical trials have shown that glucagon-like peptide-1 receptor agonists (GLP-1 RAs) cause significant weight loss and reduce cardiovascular events in patients with type 2 diabetes (T2D). Black patients have a disproportionate burden of obesity and cardiovascular disease and have a higher rate of cardiovascular-related mortality. Racial and ethnic disparities in health outcomes are largely attributable to the pervasiveness of structural racism, and patients who are marginalized by racism have less access to novel therapeutics.

**Objectives:**

To evaluate GLP-1 RA uptake among a commercially insured population of patients with T2D; identify associations of race, ethnicity, sex, and socioeconomic status with GLP-1 RA use; and specifically examine its use among the subgroup of patients with atherosclerotic cardiovascular disease (ASCVD) because of the known benefit of GLP-1 RA use for this population.

**Design, Setting, and Participants:**

This was a retrospective cohort analysis using data from OptumInsight Clinformatics Data Mart of commercially insured adult patients with T2D (with or without ASCVD) in the US. Data from October 1, 2015, to June 31, 2019, were included, and the analyses were performed in July 2020. We estimated multivariable logistic regression models to identify the association of race, ethnicity, sex, and socioeconomic status with GLP-1 RA use.

**Main Outcome and Measure:**

A prescription for a GLP-1 RA.

**Results:**

Of the 1 180 260 patients with T2D (median [IQR] age, 69 [59-76] years; 50.3% female; 57.7% White), 90 934 (7.7%) were treated with GLP-1 RA during the study period. From 2015 to 2019, the percentage of T2D patients treated with an GLP-1 RA increased from 3.2% to 10.7%. Among patients with T2D and ASCVD, use also increased but remained low (2.8%-9.4%). In multivariable analyses, lower rates of GLP-1 RA use were found among Asian (aOR, 0.59; 95% CI, 0.56-0.62), Black (adjusted odds ratio [aOR] 0.81; 95% CI, 0.79-0.83), and Hispanic (aOR, 0.91; 95% CI, 0.88-0.93) patients with T2D. Female sex (aOR, 1.22; 95% CI, 1.20-1.24) and higher zip code–linked median household incomes (>$100 000 [OR, 1.13; 95% CI, 1.11-1.16] and $50 000-$99 999 [OR, 1.07; 95% CI, 1.05-1.09] vs <$50 000) were associated with higher GLP-1 RA use. These results were similar to those found among patients with ASCVD.

**Conclusions and Relevance:**

In this cohort study of US patients with T2D, GLP-1 RA use increased, but remained low overall for treatment of T2D, particularly among patients with ASCVD who are likely to derive the most benefit. Asian, Black, and Hispanic patients and those with low income were less likely to receive treatment with a GLP-1 RA. Strategies to lower barriers to GLP-1 RA use, such as lower cost, are needed to prevent the widening of well-documented inequities in cardiovascular disease outcomes in the US.

## Key Points

**Question:**

Are there inequities in glucagon-like peptide-1 receptor agonist (GLP-1 RA) use based on race, ethnicity, sex, and socioeconomic status among patients with diabetes in the US?

**Findings:**

This 5-year cohort study of 1 180 260 commercially insured patients with diabetes in the US found that GLP-1 RA use increased but use remained low. Lower rates of GLP-1 RA use were found among Asian, Black, and Hispanic individuals and among those with lower household income; results were similar among patients with diabetes who also had cardiovascular disease.

**Meaning:**

The findings of this cohort study suggest that racial, ethnic, and socioeconomic inequities are present in access to GLP-1 RA, a medication with established benefits for improved cardiovascular outcomes in patients with diabetes.

## Teaser

This cohort study examines the racial, ethnic, sex, and socioeconomic inequities associated with use of glucagon-like peptide-1 receptor agonists in US patients with type 2 diabetes.

## Introduction

In the US, cardiovascular disease is the leading cause of mortality and morbidity among patients with type 2 diabetes (T2D).^1^ Glucagon-like peptide-1 receptor agonists (GLP-1 RAs), a recommended treatment option for glycemic control in patients with diabetes, have recently emerged as a cardioprotective therapy.^2^ Multiple large randomized clinical trials^3,4,5^ have shown GLP-1 RAs prevent cardiovascular events among patients with T2D, particularly patients with established atherosclerotic cardiovascular disease (ASCVD). These data have prompted a paradigm shift to utilize these agents not only for glycemic control, but for cardiovascular risk reduction. The American Diabetes Association updated guidelines and the American College of Cardiology expert consensus statement^6^ now recommend a GLP-1 RA with demonstrated cardiovascular benefit for patients with diabetes and established or at very high risk for ASCVD.

There are substantial disparities in diabetes prevalence, complications, and death rates. Black and Hispanic patients have a disproportionate burden of T2D.^7^ Black patients have higher diabetes complication rates, such as cardiovascular disease, and cardiovascular mortality rates are highest among Black patients.^8,9,10^ However, inequitable quality of care by race and ethnicity is a well-documented phenomenon in the US.^11^

Cardiovascular therapeutics with proven benefit are underused among Black and Hispanic patients, even among those who are commercially insured.^12,13,14,15^ In addition, there is decreased adoption of novel cardiovascular therapies among female patients and patients of low socioeconomic status. The objectives of this study were to evaluate GLP-1 RA uptake among a commercially insured population of patients with T2D; identify associations of race, ethnicity, sex, and socioeconomic status with GLP-1 RA use; and specifically examine GLP-1 RA use among the subgroup of patients with ASCVD because of its known benefit for this population.

## Methods

This retrospective cohort study was classified as exempt by the University of Pennsylvania Institutional Review Board. Informed consent was waived because the study used only deidentified data. The study followed the Strengthening the Reporting of Observational Studies in Epidemiology (STROBE) reporting guidelines.

### Study Data

Data were obtained from the OptumInsight Clinformatics Data Mart database (Optum Inc), a large administrative private payer claims database of recipients of commercial health insurance and Medicare Advantage health plans. This database consists of inpatient, outpatient, and pharmacy claims of more than 17 million patients annually from all 50 US states. Patient demographic variables, including age, sex, and race and ethnicity are collected by OptumInsight for each individual member at enrollment. The available race and ethnicity categories are Asian, Black, Hispanic ethnicity (all races), White, and other/unknown. Socioeconomic data, including median household income, are available through zip (postal) code–linked enrollment data from the US Census Bureau. Mean number of outpatient visits to a cardiologist or endocrinologist per 12-month period after cohort entry through the end of available data (June 31, 2019) was determined based on having an office visit with a cardiologist or endocrinologist with evaluation and management CPT codes 99201 to 99205 or 99211 to 99215. All prescription claims for GLP-1 RA during the study period, including all formulations of albiglutide, dulaglutide, exenatide, exenatide extended-release, liraglutide, lixisenatide, and semaglutide, were extracted.

### Study Cohort

We identified adult (age, ≥18 years) patients with a diagnosis of T2D based on the *International Statistical Classification of Diseases, Tenth Revision, Clinical Modification (ICD-10-CM)* codes E11.0, E11.1, and E11.9 from October 1, 2015, to December 31, 2018, to allow for 6 months of continuous enrollment and prescription of therapy after a diagnosis was made, given that data were available through June 31, 2019. This study period encompassed the time period when the cardiovascular benefits of GLP-1 RA use were clearly known and there was level 1 evidence of these benefits available.^3,4,5^ To ensure adequate diagnostic accuracy, patients had to have a diabetes diagnosis coded 2 or more times on different dates in either an inpatient and/or outpatient setting. Each patient entered the cohort on the date of their second diabetes diagnostic code and then were evaluated for a GLP-1 RA prescription claim for albiglutide, dulaglutide, exenatide, exenatide extended-release, liraglutide, lixisenatide, or semaglutide. The primary outcome of interest was 1 prescription for a GLP-1 RA filled at any point during the study period for each individual patient, beginning with the date of the second diabetes diagnostic code through the end of the available data (June 31, 2019).

Patients who did not have continuous insurance enrollment for 1 year or more before and 6 months or more after study entry were excluded to ensure that comorbidities, clinical data, and prescription claims could be accurately captured. In addition, patients without any pharmacy claims for medication for 1 year prior to the study period were excluded to ensure that medication use for each patient was accurately being captured in the study data. Comorbidities were evaluated from the earliest available patient data to cohort entry.

### Statistical Analysis

We divided patients into treated and not treated with a GLP-1 RA during the study period. For each group, summary statistics for patient characteristics are presented as medians (IQRs) or means (SDs) for continuous data, and as total number and percentage for categorical data. Continuous variables were compared using the Student *t* test, and categorical variables were compared using the χ^2^ test. We described the proportion of patients using a GLP-1 RA during the entire study period and for each year. We repeated this analysis for Asian, Black, and Hispanic patients, and for those with ASCVD. For those who filled a GLP-1 RA prescription, we determined the median (IQR) of the 30-day prescription copayment for the first filled prescription.

To assess the relationship of race and ethnicity with GLP-1 RA use, we estimated multivariable logistic regression models with filled GLP-1 RA prescription as the dependent variable. The independent variables included age, sex, race or ethnicity (Asian, Black, Hispanic, White), region of residence, zip code–linked household income, health insurance subset (commercial-only or Medicare Advantage, which provides Medicare benefits through commercial insurers), hyperlipidemia, coronary artery disease, cerebrovascular disease, chronic kidney disease, kidney failure or end-stage kidney disease, hypertension, obesity, peripheral vascular disease, heart failure with reduced ejection fraction (HFrEF), heart failure with preserved ejection fraction (HFpEF), the number of Elixhauser comorbidities,^16^ number of visits to a cardiologist per 12-month period, number of visits to an endocrinologist per 12-month period, insulin use, and metformin use. We repeated this analysis in the subgroup of patients with a diagnosis of ASCVD based on the *ICD-10-CM* codes (eTable in the Supplement). Patients with missing data for any 1 of the aforementioned covariates were not included in the multivariable analyses.

Estimated adjusted odds ratios (aORs) are reported with 95% CIs. Statistical analyses were performed using SAS, version 9.4 (SAS Institute Inc). All statistical testing was 2-tailed with *P* values < .05 defined as being statistically significant.

## Results

**Figure 1.**
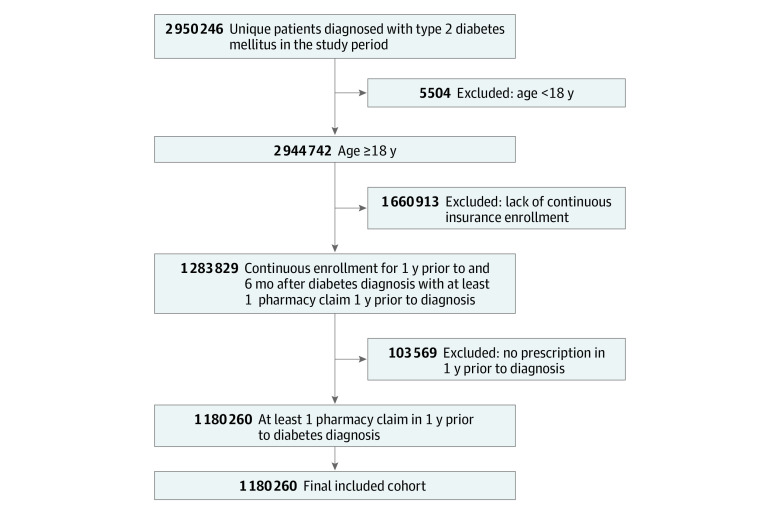
CONSORT Diagram of Applied Exclusion Criteria and Final Study Cohort

Of the 1 180 260 patients with T2D (median [IQR] age, 69 [59-76] years; 594 088 women [50.3%]) who met inclusion criteria (Figure 1), 52 349 were Asian (4.4%); 146 861, Black (12.4%); 173 561, Hispanic (14.7%); and 681 579, White (57.7%). During the study period, 92.3% (1 089 326 patients) had not been prescribed a GLP-1 RA and 7.7% (90 934 patients) had filled a GLP-1 RA prescription. The zip code–linked median household income was less than $50 000 for 31.3% (369 474 patients) of the cohort and $100 000 or more for 209 200 patients (17.7%). Baseline demographic, socioeconomic, and clinical differences between those who were prescribed a GLP-1 RA vs those who were not are summarized in Table 1.

**Table 1.** Baseline Characteristics of Patients With Diabetes, by Glucagon-Like Peptide 1 Receptor Agonists (GLP-1 RA) Prescription Status

Characteristic	No. (%)		*P* value
	No prescription (n = 1 089 326)	GLP-1 RA use (n = 90 934)	
Age, median (IQR)	70 (60-77)	59 (51-68)	<.001
Sex			
Female	547 292 (50.2)	46 796 (51.5)	<.001
Male	541 903 (49.7)	44 130 (48.5)	
Race and ethnicity			
Asian	49 995 (4.6)	2354 (2.6)	<.001
Black	135 962 (12.5)	10 899 (12.0)	
Hispanic	160 191 (14.7)	13 370 (14.7)	
White	624 643 (57.3)	56 936 (62.6)	
Other/unknown^a^	118 535 (10.9)	7375 (8.1)	
Region of residence			
Midwest	220 717 (20.3)	20 693 (22.8)	<.001
Northeast	141 677 (13.0)	7634 (8.4)	
South	470 746 (43.2)	45 610 (50.2)	
West	253 585 (23.3)	16 856 (18.5)	
Unknown	2601 (0.2)	141 (0.2)	
Zip code–linked median household income, $			
<50 000	344 405 (31.6)	25 069 (27.6)	<.001
50 000-99 999	324 033 (29.7)	27 673 (30.4)	
≥100 000	188 165 (17.3)	21 035 (23.1)	
Unknown	232 723 (21.4)	17 157 (18.9)	
Health insurance subtype			
Commercial	341 441 (31.3)	53 682 (59.0)	<.001
Medicare Advantage	747 885 (68.7)	37 252 (41.0)	
Comorbidities			
Dyslipidemia	950 226 (87.2)	80 589 (88.6)	<.001
Cerebrovascular disease	247 473 (22.7)	13 986 (15.4)	<.001
Coronary artery disease	114 750 (10.5)	6987 (7.7)	<.001
Coronary artery bypass graft surgery	17 355 (1.6)	1218 (1.3)	<.001
Chronic kidney disease	270 757 (24.9)	17 326 (19.1)	<.001
End-stage kidney disease	18 368 (1.7)	668 (0.7)	<.001
Obesity	344 707 (31.6)	43 639 (48.0)	<.001
Hypertension	926 911 (85.1)	76 906 (84.6)	<.001
Peripheral vascular disease	240 657 (22.1)	12 489 (13.7)	<.001
HFrEF	57 971 (5.3)	2801 (3.1)	<.001
HFpEF	55 496 (5.1)	2693 (3.0)	<.001
Elixhauser comorbidities, No.			
0-1	216 669 (19.9)	18 833 (20.7)	<.001
2-3	375 720 (34.5)	35 847 (39.4)	
4-6	316 300 (29.0)	26 202 (28.8)	
≥7	180 637 (16.6)	10 052 (11.1)	
Medications			
Metformin	508 259 (46.7)	58 602 (64.4)	<.001
Insulin	174 724 (16.0)	35 624 (39.2)	<.001
Endocrinologist visit(s), No. per 12 mo			
0	992 831 (91.1)	63 760 (70.1)	<.001
1	43 234 (4.0)	9616 (10.6)	
>1	53 261 (4.9)	17 558 (19.3)	
Cardiologist visit(s), No. per 12 mo			
0	778 972 (71.5)	64 124 (70.5)	<.001
1	143 662 (13.2)	13 873 (15.3)	
>1	166 692 (15.3)	12 937 (14.2)	

Abbreviations: HFpEF, heart failure with preserved ejection fraction; HFrEF, heart failure with reduced ejection fraction.

^a^ Racial and ethnic category of other/unknown includes patients who identify as a race other than Asian, Black, Hispanic, or White.

**Figure 2.**
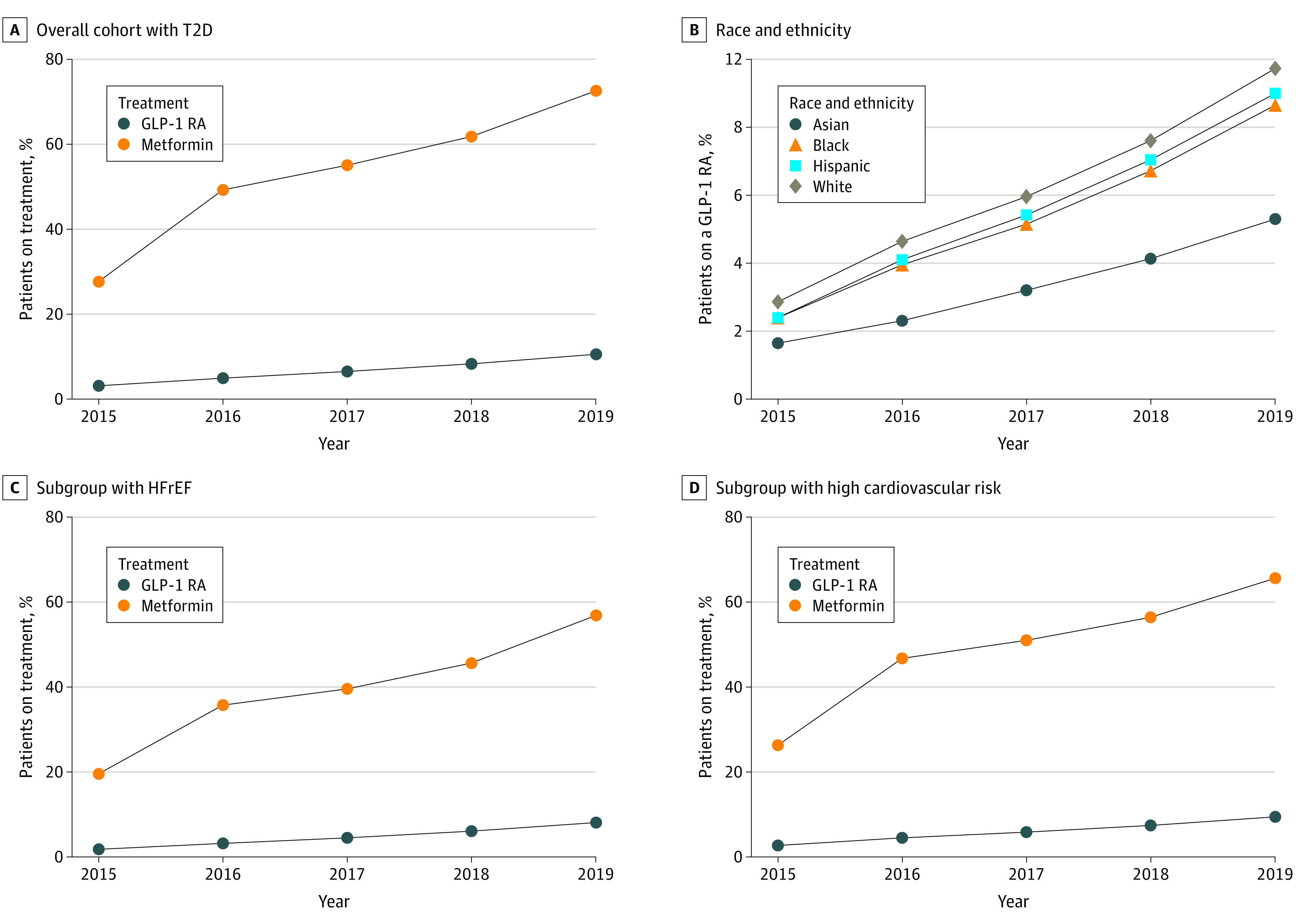
Accumulated Rates of GLP-1 RA Use Among a Cohort of Patients With T2D, by Race and Ethnicity and by Subgroup, 2015 to 2019 A, Percentage of overall cohort being treated with GLP-1 RA vs metformin demonstrates low use of a GLP-1 RA. B, Race and ethnicity of patients using a GLP-1 RA demonstrates higher rates among White patients and lowest rate among Asian patients. C, Among patients who also have cardiovascular disease, GLP-1 RA use is low. D, Among patients at higher risk of a cardiovascular event or disease, GLP-1 RA use is low. GLP-1 RA denotes glucagon-like peptide-1 receptor agonist; HFrEF, heart failure with reduced ejection fraction; and T2D, type 2 diabetes.

The proportion of patients with diabetes being treated with a GLP-1 RA increased from 3.2% in 2015 to 10.7% in 2019 (Figure 2A) overall; from 2.0% to 6.4% among Asian patients, 2.9% to 10.4% among Black patients, 2.9% to 10.8% among Hispanic patients, and 3.6% to 11.7% among White patients (Figure 2B). Among those who also had ASCVD (815 309 patients), the GLP-1 RA use increased from 2.8% to 9.4%. Patients who filled a GLP-1 RA prescription, had a median (IQR) 30-day copayment of $40.00 (IQR, $8.35–$60.00).

In multivariable analyses (Table 2), female sex was associated with higher odds of GLP-1 RA use (aOR, 1.22; 95% CI, 1.20-1.24). Compared with White individuals, Asian (aOR, 0.59; 95% CI, 0.56-0.62), Black (aOR, 0.81; 95% CI, 0.79-0.83), and Hispanic (aOR, 0.91; 95% CI, 0.88-0.93) individuals had lower GLP-1 RA use. Higher annual median household incomes (>$100 000 and $50 000-$99 999) were associated with higher GLP-1 RA use (aOR, 1.13 [95% CI, 1.11-1.16] and 1.07 [95% CI, 1.05-1.09], respectively) compared with lower income (<$50 000).

**Table 2.** Factors Associated With Glucagon-Like Peptide 1 Receptor Agonists (GLP-1 RA) Use Among All Patients on Multivariable Analysis

Characteristic	Odds ratio (95% CI)	*P* value
Age, y	0.97 (0.97-0.97)	<.001
Sex		
Female	1.22 (1.20-1.24)	<.001
Male	1 [Reference]	
Race and ethnicity		
Asian	0.59 (0.56-0.62)	<.001
Black	0.81 (0.79-0.83)	<.001
Hispanic	0.91 (0.88-0.93)	<.001
White	1 [Reference]	
Region of residence		
Midwest	1.01 (0.98-1.04)	.44
Northeast	0.79 (0.76-0.81)	<.001
South	1.17 (1.14-1.20)	<.001
West	1 [Reference]	
Zip code–linked median household income		
<$50 000	1 [Reference]	
$50 000-$99 999	1.07 (1.05-1.09)	<.001
$>100 000	1.13 (1.11-1.16)	<.001
Insurance subtype		
Commercial	1.53 (1.50-1.57)	<.001
Medicare Advantage	1 [Reference]	
No. of Elixhauser comorbidities	0.93 (0.93-0.93)	<.001
Dyslipidemia	1.55 (1.51-1.59)	<.001
Coronary artery disease	0.95 (0.92-0.98)	.001
Cerebrovascular disease	0.96 (0.93-0.98)	<.001
Chronic kidney disease	1.26 (1.23-1.29)	<.001
End-stage kidney disease	0.49 (0.44-0.54)	<.001
Obesity	1.72 (1.69-1.75)	<.001
Hypertension	1.49 (1.45-1.53)	<.001
Peripheral vascular disease	1.00 (0.97-1.03)	.95
HFrEF	0.83 (0.79-0.88)	<.001
HFpEF	0.87 (0.83-0.92)	<.001
Metformin use	1.88 (1.85-1.91)	<.001
Insulin use	2.65 (2.60-2.69)	<.001
Cardiologist visit(s) per 12 mo		
0	1 [Reference]	
1	1.19 (1.16-1.22)	<.001
>1	1.16 (1.13-1.19)	<.001
Endocrinologist visit(s) per 12 mo		
0	1 [Reference]	
1	2.26 (2.20-2.33)	<.001
>1	3.14 (3.07-3.22)	<.001

Abbreviations: HFpEF, heart failure with preserved ejection fraction; HFrEF, heart failure with reduced ejection fraction.

Coronary artery disease (aOR, 0.95; 95% CI, 0.92-0.98) and cerebrovascular disease (aOR, 0.96; 95% CI, 0.93-0.98) were both independently associated with lower GLP-1 RA use. Having more Elixhauser comorbidities was associated with lower GLP-1 RA use (aOR, 0.93; 95% CI, 0.93-0.93).

Having more visits to an endocrinologist per 12-month period was independently associated with increased GLP-1 RA use: either 1 visit (aOR, 2.26; 95% CI, 2.20-2.33) or more than 1 visit (aOR, 3.14; 95% CI, 3.07-3.22) compared with no visits. Similarly, having more visits to a cardiologist per 12-month period was independently associated with increased GLP-1 RA use: either 1 visit (aOR, 1.19; 95% CI, 1.16-1.22) or more than 1 visit (aOR, 1.16; 95% CI, 1.13-1.19) compared with no visits.

### Subgroup Analysis

Factors associated with GLP-1 RA use among patients with T2D and ASCVD on multivariable analyses are shown in Table 3. Female sex was associated with higher GLP-1 RA use (aOR, 1.18; 95% CI, 1.15-1.20). Asian patients (aOR, 0.69; 95% CI, 0.65-0.73), Black patients (aOR, 0.82; 95% CI, 0.79-0.85), and Hispanic patients (aOR, 0.94; 95% CI, 0.91-0.96) had less GLP-1 RA use than White patients. Higher median household incomes (>$100 000 and $50 000-$99 999) were also associated with more GLP-1 RA use (aOR, 1.06 [95% CI, 1.03-1.08] and 1.15 [95% CI, 1.11-1.18], respectively) compared with lower income ( <$50 000).

**Table 3.** Factors Associated With GLP-1 RA Use Among Patients With ASCVD on Multivariable Analysis

Characteristic	Odds ratio (95% CI)	*P* value
Age, y	0.96 (0.96-0.96)	<.001
Sex		
Female	1.18 (1.15-1.20)	<.001
Male	1 [Reference]	
Race and ethnicity		
Asian	0.69 (0.65-0.73)	<.001
Black	0.82 (0.79-0.85)	<.001
Hispanic	0.94 (0.91-0.96)	<.001
White	1 [Reference]	
Region of residence		
Midwest	0.97 (0.93-1.00)	.04
Northeast	0.78 (0.75-0.82)	<.001
South	1.13 (1.10-1.17)	<.001
West	1 [Reference]	
Zip code–linked median household income		
<$50 000	1 [Reference]	
$50 000-$99 999	1.06 (1.03-1.08)	<.001
$>100 000	1.15 (1.11-1.18)	<.001
Insurance subtype		
Commercial	1.42 (1.38-1.46)	<.001
Medicare Advantage	1 [Reference]	
No. of Elixhauser comorbidities	0.92 (0.92-0.93)	<.001
Dyslipidemia	1.57 (1.51-1.63)	<.001
Coronary artery disease	0.93 (0.90-0.96)	.001
Cerebrovascular disease	0.96 (0.93-0.98)	.0002
Chronic kidney disease	1.29 (1.26-1.33)	<.001
End-stage kidney disease	0.45 (0.41-0.50)	<.001
Obesity	1.73 (1.70-1.77)	<.001
Hypertension	1.43 (1.38-1.49)	<.001
Peripheral vascular disease	0.99 (0.96-1.02)	.57
HFrEF	0.83 (0.79-0.87)	<.001
HFpEF	0.88 (0.84-0.93)	<.001
Metformin use	1.92 (1.88-1.96)	<.001
Insulin use	2.81 (2.74-2.87)	<.001
Cardiologist visit(s) per 12 mo		
0	1 [Reference]	
1	1.15 (1.12-1.18)	<.001
>1	1.15 (1.12-1.18)	<.001
Endocrinologist visit(s)/12 mo		
0	1 [Reference]	
1	2.13 (2.06-2.21)	<.001
>1	3.08 (2.99-3.17)	<.001

Abbreviations: ASCVD, atherosclerotic cardiovascular disease; GLP-1 RA, glucagon-like peptide-1 receptor agonist; HFpEF, heart failure with preserved ejection fraction; HFrEF, heart failure with reduced ejection fraction.

## Discussion

In this study, we found low use of GLP-1 RAs, including among patients with T2D and ASCVD. Despite that 100% of this population was commercially insured, this is the first study, to our knowledge, to demonstrate notable racial, ethnic, and socioeconomic inequities in GLP-1 RA use. Asian, Black, and Hispanic individuals had lower use of GLP-1 RA, while higher household income was independently associated with higher use. These inequities were also present among patients with T2D who also had ASCVD.

Structural racism, defined as the differential distribution of goods, services, and opportunities of society based on race,^17^ is a major barrier to achieving health equity.^18^ Similar to sodium-glucose cotransporter 2 (SGLT2) inhibitor use,^12^ independently associated lower use of GLP-1 RA was found among Asian and Black individuals. Additionally, Hispanic individuals also had lower GLP-1 RA use. Patients of racial and ethnic minority groups consistently have inequitable access to guideline-based therapeutics that improve cardiovascular disease burden and outcomes, despite often experiencing a disproportionately higher rate of these conditions.^10,19^ Often, socioeconomic status and lack of health insurance are blamed for racial and ethnic inequities in health care and health outcomes.^20,21^ However, the racial and ethnic disparities in GLP-1 RA use demonstrated by the present study persisted after adjustment not only for clinical factors, but also for engagement with specialty care and socioeconomic status, and were in the setting of a 100% commercially insured population. Therefore, these results reveal biases in health care delivery that must be rectified.

While the diabetes-related risk for coronary heart disease has declined among White patients since 1990, it has doubled among Black patients.^9^ Therefore, a better understanding of the barriers to GLP-1 RA use among this population and other marginalized groups is needed. Inequitable uptake must be addressed to prevent the widening of racial and ethnic disparities in cardiovascular disease and outcomes in the US.^8^

As with other novel cardiovascular therapies,^12,13,15^ including SGLT2 inhibitors, socioeconomic status was independently associated with lower GLP-1 RA use. Despite adequate prescription drug insurance, among patients with Medicare Part D coverage, the median estimated annual out-of-pocket cost for liraglutide is $2447.^22^ We found that among patients who filled a prescription for GLP-1 RA, the median 30-day copayment was $40; these findings suggest that the cost is likely prohibitive and may be worsening inequities by potentially encouraging utilization of inexpensive medications, which may have no cardiac benefit, among marginalized patient groups. In fact, primary care physicians and endocrinologists cite cost and unapproved prior authorizations as the major barriers to prescribing these agents.^23^ Additionally, although we adjusted for median household income, cost is likely to contribute to racial and ethnic inequities given this country’s wealth disparities and the inability of some groups to afford out-of-pocket costs.^24^

Interestingly, unlike with SGLT2 inhibitor use and other cardiovascular therapeutics,^12,25^ female sex was associated with higher GLP-1 RA use. Previous studies have shown^26^ that, among diabetic patients, men may be more comprehensively treated in regard to coronary heart disease risk. It is unclear why GLP-1 RA use was higher among women in the present study; it may reflect the well-documented higher rates of contact with the health care system among female patients.^27^ In addition to the cardiovascular benefits, GLP-1 RAs have also been shown to cause significant and sustained weight loss.^28^ It is possible that female patients with T2D may engage more with a nutritionist and may be more likely to seek pharmacotherapy for medical management of obesity, which may include counseling for GLP-1 RA use.^29^

Notably, Asian patients had the lowest rates of GLP-1 RA use and more than 40% lower odds of GLP-1 RA prescription. Barriers to accessing care, less patient-centered interactions by practitioners, and biases in care delivery have been well-documented among Asian patients and likely play a role in the inequitable use of GLP-1 RA in this population.^30,31^ Among patients with T2D, Asian patients have the lowest body mass index compared with other races and ethnicities,^32^ a difference that may influence prescribing practices. However, these findings are consistent with previously documented inequitable SGLT2 inhibitor use among Asian and Black patients^12^ and reflect the pervasive inequity of the US health care system for patients who are not White.

Similar to the findings of prior studies of T2D outpatient registries,^33,34,35,36,37^ the results of the present study confirm low use of GLP-1 RA, only slightly higher among a commercially insured cohort. Despite the multiple randomized clinical trials conducted since 2015 that demonstrate improved cardiovascular outcomes with GLP-1 RA use among patients with ASCVD,^3,4,5^ the rate of use among this subgroup remained low through 2019 (9.4%)—lower than among the overall diabetes cohort (10.7%). The updated guidelines from the American Diabetes Association and the statement from the American College of Cardiology strongly recommend GLP-1 RA use for patients with T2D who already have or are at high risk for cardiovascular disease.^2,6^ The results of this study suggest that GLP-1 RA and similar agents have not yet been adopted as a strategy for broader cardiovascular risk reduction. In fact, coronary artery disease and cerebrovascular disease were associated with lower GLP-1 RA use. Cardiologists’ prescription of GLP-1 RA has previously been shown to be minimal, even among patients with cardiovascular conditions.^36^ In our cohort, a visit with a cardiologist was associated with higher GLP-1 RA use and having a visit with an endocrinologist was among the strongest predictors of GLP-1 RA use (>3 times the odds of prescription). Yet, only a small percentage of patients with T2D receive care from an endocrinologist^38^ and patients with cardiovascular disease are much more likely to have a cardiologist than an endocrinologist on their care team.^39^ Therefore, along with other traditional agents, such as statins, cardiologists must start viewing prescription of these agents as part of their cardiac risk reduction armamentarium.^23^ In addition, given the demonstrated inequities in accessing cardiology care,^40,41^ it is essential that barriers be decreased and that knowledge and comfort be increased to facilitate prescribing of these agents by primary care practitioners to all patients with T2D and ASCVD risk factors, with special attention to marginalized groups of patients.

### Limitations

This study had several limitations. The factors and clinical decision-making that drive the decision to initiate a certain therapy, such as GLP-1 RA can be complex and are not well-characterized in an administrative database. Given that most GLP-1 RAs are injectable, there may be residual confounding by patient preference. Patient self-advocacy might also influence treatment decisions, if White patients more frequently advocate for this therapy because of their greater awareness of its benefits. Racial differences in self-advocacy have been previously observed and must be contextualized in a health care system with historic and ongoing discrimination against racial and ethnic minority groups in the US.^42^ Additionally, a practitioner’s preference, knowledge of guidelines and benefits, and level of comfort with any therapy influence its use; disparities may be driven, in part, by where a patient seeks care. The database used for this study (OptumInsight Clinformatics Data Mart) captured prescriptions filled at pharmacies; thus, we were unable to distinguish between barriers in practitioners’ prescription patterns vs barriers in prescription fulfillment. The demonstrated differences may be partly associated with prescription abandonment at the pharmacy given the high copayment cost; however, we were unable to determine whether this, practitioner bias, or other barriers explain the study findings. Additional aspects of benefit design may mediate some of the findings, but we are unable to characterize the extent to which this may contribute because copayment data are not available for patients who did not fill their prescription for this therapy. There are contraindications to and adverse effects of GLP-1 RA therapy, which may have contributed to the results. We adjusted for visits to a cardiologist and/or endocrinologist, but the database did not allow us to evaluate the type of practice or health care professional prescribing the therapy, which limited our understanding of how differences in access to specialty care contributed to the findings. There were missing data at baseline, including data on race, ethnicity, and median household income. More granular socioeconomic status data that may also influence a patient’s ability to fill a prescription were also missing from the database. Additionally, the database was unable to fully capture how race and ethnicity operate in a broader socioeconomic and sociopolitical context, the complete effects of structural racism, a long and continued history of mistreatment, and levels of discrimination, which all affect how patients of racial and ethnic minority groups navigate the US health care system and how they receive care.^11,18^

## Conclusions

This retrospective cohort study of US patients with T2D found low rates of GLP-1 RA use, even among patients who also had ASCVD. We found an independent association of lower GLP-1 RA use among Asian, Black, and Hispanic patients and among those with lower zip code–linked household income, with a similar pattern of inequitable use among patients who also had ASCVD. Implementation of strategies to ensure more equitable use of GLP-1 RA therapy is warranted.

## Notes

Supplement. **eTable**. Included ICD Codes for Atherosclerotic Cardiovascular Disease
